# Identification of novel putative causative genes and genetic marker for male sterility in Japanese cedar (*Cryptomeria japonica* D.Don)

**DOI:** 10.1186/s12864-018-4581-5

**Published:** 2018-04-23

**Authors:** Kentaro Mishima, Tomonori Hirao, Miyoko Tsubomura, Miho Tamura, Manabu Kurita, Mine Nose, So Hanaoka, Makoto Takahashi, Atsushi Watanabe

**Affiliations:** 1 Forest Tree Breeding Center, Forestry and Forest Products Research Institute, Forest Research and Management Organization, 3809-1 Ishi, Juo, Hitachi, Ibaraki, 319-1301 Japan; 2 Department of Forest Environmental Sciences, Faculty of Agriculture, Kyushu University, 6-10-1 Hakozaki, Higashi-ku, Fukuoka, 812-8581 Japan

**Keywords:** *Cryptomeria japonica*, Male sterility, Marker-assisted selection, SNP discovery

## Abstract

**Background:**

Japanese cedar (*Cryptomeria japonica*) is an important tree for Japanese forestry. Male-sterile marker development in Japanese cedar would facilitate selection of male-sterile plus trees, addressing the widespread social problem of pollinosis and facilitating the identification of heterozygotes, which are useful for breeding.

**Results:**

This study used next-generation sequencing for single-nucleotide polymorphism discovery in libraries constructed from several organs, including male-sterile and male-fertile strobili. The single-nucleotide polymorphisms obtained were used to construct a high-density linkage map, which enabled identification of a locus on linkage group 9 strongly correlated with male-sterile trait. Expressed sequence tags corresponding to 11 marker loci from 5 isotigs were associated with this locus within 33.4-34.5 cM. These marker loci explained 100% of the phenotypic variation. Several homologs of these sequences are associated with male sterility in rice or *Arabidopsis*, including a pre-mRNA splicing factor, a DEAD-box protein, a glycosyl hydrolase, and a galactosyltransferase. These proteins are thus candidates for the causal male-sterile gene at the *ms-1* locus. After we used a SNaPshot assay to develop markers for marker-assisted selection (MAS), we tested F_2_ progeny between male-sterile and wild-type plus trees to validate the markers and extrapolated the testing to a larger plus-tree population. We found that two developed from one of the candidates for the causal gene were suitable for MAS.

**Conclusions:**

More than half of the ESTs and SNPs we collected were new, enlarging the genomic basis for genetic research on Japanese cedar. We developed two SNP markers aimed at MAS that distinguished individuals carrying the male-sterile trait with 100% accuracy, as well as individuals heterozygous at the male-sterile locus, even outside the mapping population. These markers should enable practical MAS for conifer breeding.

**Electronic supplementary material:**

The online version of this article (10.1186/s12864-018-4581-5) contains supplementary material, which is available to authorized users.

## Background

Japanese cedar (*Cryptomeria japonica*) is a coniferous species endemic to Japan. As a major forestry species with a long afforestation history [1] and excellent attributes, Japanese cedar occupies 4.5 million ha or 44% of artificial forests in Japan. Yearly, 17 million Japanese cedar seedlings are supplied as planting stock for forestation [2]. A breeding program was launched in the 1950s, and more than 3700 Japanese cedar plus trees have been selected and evaluated in the program. However, pollen of the species produces allergens affecting about 30% of the Japanese population [3]. Therefore, not only forestry traits such as growth and wood properties but also fecundity of male strobili are targets of the breeding programs. Four male-sterile causative loci from 23 male-sterile individuals have been classified according to anomalies during pollen formation [4, 5]. Large numbers of expressed sequence tags (ESTs) in male strobili and simple sequence repeats (SSRs) have been identified for linkage maps, allowing efficient use of male sterility in tree breeding [6–16]. The *ms-1*, *ms-2, ms-3, and ms-4* loci were identified on different linkage groups (LG9, LG5, LG1, and LG4 respectively) in a high-density map [17–21], and adjacent markers were developed for marker-assisted selection (MAS) [18, 21]. The accuracy of the markers was 96.0 to 98.5% within the mapping population [18], but the markers cannot be exploited outside of this population for screening of male-sterile gene heterozygotes or cryptic carriers, which are important breeding material. Additionally, the causative genes have not been discussed [17–21].

Next-generation sequencing technology has been expanded to non-model organisms, capturing large-scale variation covering the whole genome. Although genome information for Japanese cedar is limited [22, 23], approaches utilizing genome-wide information have become more important for accelerating tree breeding of coniferous species. Draft genome sequences have been reported in Norway spruce (*Picea abies*) and loblolly pine (*Pinus teada*) [24–26], with many studies of genomic selection and genome-wide association [27–32]. The relatively short linkage disequilibrium of Japanese cedar and a marker interval for genomic selection of more than one marker per centimorgan [33] suggests that genomic selection would be efficient.

Our objective was to discover candidate causative genes for male sterility and to develop efficient markers for MAS, facilitating breeding in Japanese cedar. We collected expressed sequence tags (ESTs) from several organs using next-generation sequencing, constructed a high-density linkage map of a massive number of single nucleotide polymorphisms (SNPs), carried out quantitative trait locus (QTL) analysis using an F_2_ population, and developed SNP markers associated with the male-sterile trait. We also validated their marker potential by screening for cryptic carriers outside of the mapping population using plus trees of Japanese cedar.

## Results and discussion

### EST collection, sequencing and de novo assembly

Two sequencing platforms, the Roche 454 and Illumina HiSeq system, were used to sequence 19 EST libraries constructed from multiple organs (Additional file 1); the sequencing and assembly results are summarized in Additional file 2. The sequence data used for the following analysis, including already reported data (cambium during the active season (DRA000525) [34] and shoots (DRA001261) [35]), were from approximately 3 million reads with an average length of 437.8 bp, amounting to 1.7 Gbp. The variation in obtained isotig number is presumably related to the sequencing read number for each library.

**Fig. 1:**
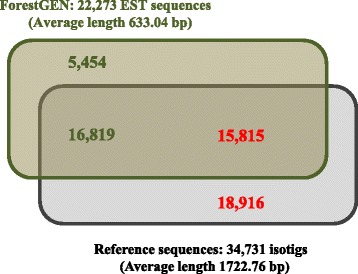
Venn diagram showing the overlap between our reference sequences and sequences in the ForestGEN database

All sequences generated from the nine libraries generated from the Roche 454 platform were assembled into 34,731 isotigs and these assembled isotigs were regarded as reference sequences. The mode and arithmetic average of isotig length were 1000 bp and 1724. 57 bp, respectively (Additional files 2 and 3). More than 99% of the isotigs were longer than 100 bp, and the median (N_50_) was 2075 bp. The depth of reference sequences ranged from 1 to 364.99, with an average of 25.18 reads/site in each isotig (Additional file 2). The size distribution of the reference sequences was compared with several model plants such as *Arabidopsis thaliana*, *Oryza sativa*, *Zea mays*, *Selaginella moellendorffii*, *Physcomitrella patens*, *Populus trichocarpa*, *P. abies*, and *P. taeda* (Additional file 3). The length of the reference sequences of Japanese cedar was at least as long as the model plants, suggesting that most of the isotigs obtained include nearly full-length gene sequence information. Our reference sequences generated from these nine libraries were BLASTN searched against EST sequences in the Japanese cedar database (ForestGEN), resulting in 15,815 sequences with significant matches in ForestGEN (Fig. 1); more than half of the sequences (18,916 isotigs) were regarded as newly collected in Japanese cedar. Thus, our effort nearly doubled the number of EST resources in Japanese cedar.

All the reference sequences generated from the nine libraries on the Roche 454 platform were compared with isotigs of each library and classified into four categories of organs (male strobili, shoots, wood and roots) (Additional file 4). The number of homologous isotigs thought to be expressed in each organ was 8234 in male strobili, 19,882 in shoots, 19,942 in wood and 16,302 in roots (Additional file 4). Though 5486 isotigs (16.3%) were common across organs, 477 isotigs (1.4%) in strobili, 3277 isotigs (9.4%) in shoots, 3573 isotigs (10.3%) in wood, and 2458 isotigs (7.1%) in roots were organ specific (Additional file 4). These results influenced the number of reads obtained; however, these distinct isotigs presumably reflect organ-specific or common functions of genes expressed in each organ.

### SNP discovery

In the process of SNP discovery, from a total of ~ 540.1 billion cleaned reads, a total of 573,795 SNPs were identified (Additional file 5) and 73,274 SNPs considered as robust for genotyping were selected as Axiom_Cj70K_ver. 1 (Additional file 1); from secondary SNP discovery, 89,993 SNPs were identified (Additional files 1 and 5).

**Fig. 2:**
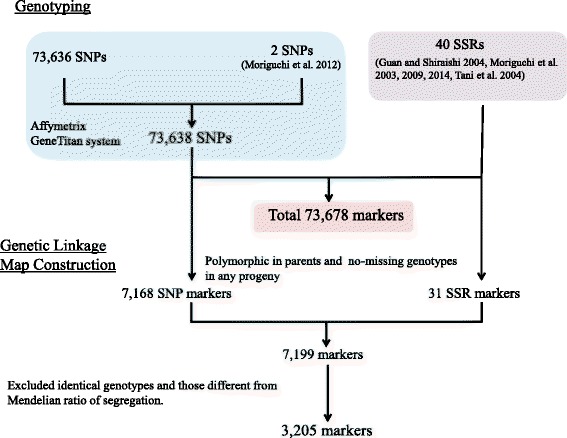
Summary of markers used and flow for map construction in this study

**Fig. 3:**
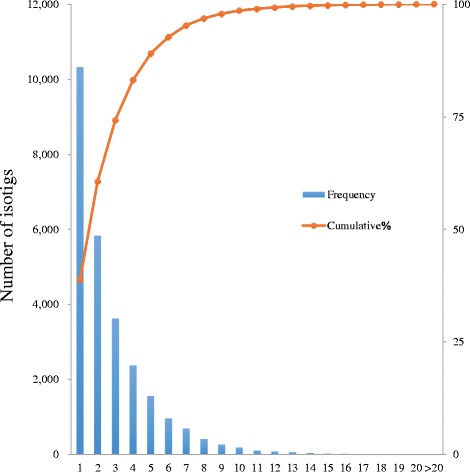
The distribution of number of SNPs per isotig and cumulative percentage of SNP

In the Axiom genotyping assay for the set of reads called Axiom_Cj70K_ver. 1, genotyping showed that 53,378 out of 73,274 SNPs obtained a reliable genotype (Hiraoka et al. unpublished) for 386 plus trees; therefore, from the genotyped SNPs and 89,993 SNPs used for secondary SNP discovery, 73,638 new SNPs obtained following removal of duplicate and undesignable SNPs and addition of 2 SNPs (cj_gSNP01452 and cj_gSNP0438) reportedly near the *ms-1* locus [17] were selected as Axiom_Cj70K_ver. 2 (Fig. 2, Additional file 1). The 73,638 SNPs based on isotigs covered 26,689 isotigs with an average number of SNPs per isotig of 2.76. Nearly 40% had one SNP per isotig and nearly 60% had two or more (Fig. 3).

### Phenotyping

In this study, the fertile and male-sterile progeny segregated at 148:42 (approximately a 3:1 ratio; chi-square test *P* > 0.01), suggesting that inheritance of the male-sterile trait is controlled by a single recessive Mendelian locus.

Developing male-sterile ‘Sosyun’ pollen has aberrant morphology in the transient phase from the tetrad to microspore stage and thereafter [16]. This phenotype resembles that of sterile trees controlled by the recessive *ms-1* locus, which is also manifested at the tetrad stage [4, 5, 17], suggesting that ‘Sosyun’ male sterility may be controlled by *ms-1*.

### Genotyping

**Fig. 4:**
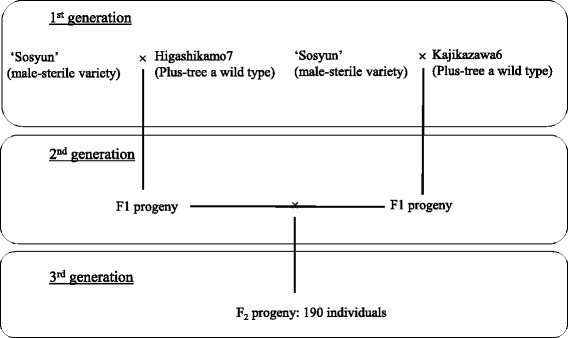
The three-generation pedigree of analyzed for linkage mapping in this study

A total of 73,638 SNP markers were used for genotyping 190 progeny from the F_2_ and their three contributing parents (Fig. 4). Out of 73,638 SNP markers, 7168 were polymorphic and categorized as poly-high resolution by the Axiom quality control criteria, segregating in F_2_ progeny in a way concordant with the genotype of their parents and grandparents, with no missing genotypes. Out of the 7168 markers and 31 SSRs, 7199 markers in total, 3205 were used to construct a genetic linkage map after excluding markers with genotypic segregation significantly different from Mendelian segregation (when markers with identical segregation pattern were included, the number rose to 6629 markers; Fig. 2, Additional file 6).

### Construction of genetic linkage map and QTL analysis

**Fig. 5:**
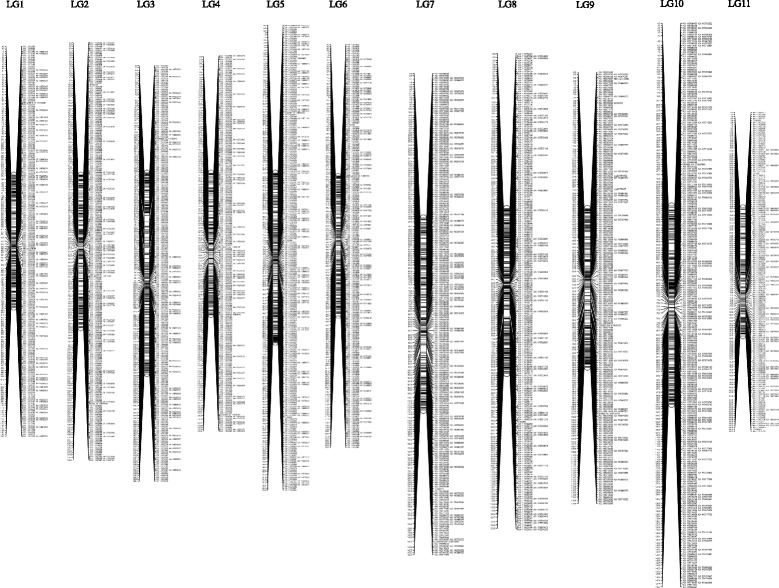
A genetic linkage map for Japanese cedar in this study. Marker names and genetic distance (Kosambi) are displayed to the right and left of the linkage group, respectively

The high-density linkage map constructed from the 3205 markers allowed assignment of 11 linkage groups, covering 1492.8 cM (mean distance between adjacent markers of 0.47 cM/marker) and consisting of 4649 expected genes (Fig. 5, Additional file 6). The total genetic distance was close to the length of the reference map (1405 cM) for Japanese cedar, and the order of anchor markers (SSRs) in the new map also corresponded to that in reference map [17]. The average interval between markers in the new map was similar to that in reported reference maps for this species (1.1 cM/marker [17], 0.49 cM/marker [20]) and in map for other conifers (0.58-0.62 cM/marker in *P. taeda,* [36, 37], 0.6 cM/marker in a consensus map for *P. taeda* and *Pinus elliottii* [38]*,* 0.93 cM/marker in *Pinus pinaster* [39], 0.92 cM/marker in a consensus map for *Picea glauca* and *Pinus marina* [40]).

**Fig. 6:**
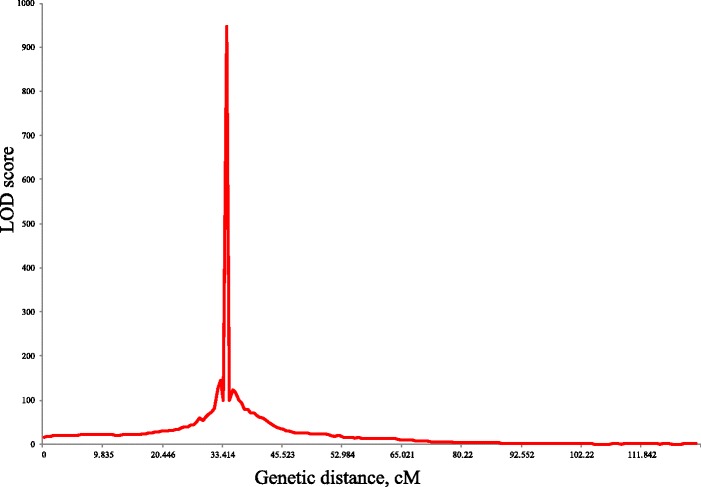
The true LOD values for LG9

We identified one notable QTL peak for male sterility. An LOD score of the strongest QTL peak was observed for the trait between 33.4 and 34.5 cM on linkage group 9 (LG9; Fig. 6), which overlapped with the reported QTL region for *ms-1* [17, 18]. Eleven marker loci located to five isotigs, listed in Table 1, were found in the region. These loci were able to discriminate male-sterile progeny in the F_2_ mapping population with an accuracy of 100%, higher than markers reported previously (96-98.5%) [17, 18], suggesting that these loci may be within the causative gene or tightly linked to the male sterility gene of ‘Sosyun’.

**Table 1:** QTLs for male-sterile traits detected in this study

Locus (marker name)	Linkage group	Position (cM)	LOD score	Phenotypic variation explained	EST name	Sequence position	SNP	Segregation type	Description	Length of EST (bp)	E-value	Homologous Arabidopsis locus
AX-115676114	9	33.414	99.99	100.0	MF_BC_CLC_contig_46230	309	A/G	lmxll	SNARE associated Golgi protein family	530	4.29	AT4G14950.2
AX-115695236	9	33.414	99.99	100.0	reCj11611	1639	A/G	lmxll	Glycosyl hydrolase family 47 protein	2558	0.00	AT5G43710.1
AX-115678020	9	33.414	99.99	100.0	reCj19250	1927	A/G	hkxhk	PRH75 DEAD box RNA helicase (PRH75)	2841	0.00	AT5G62190.1
AX-115675027	9	33.414	99.99	100.0	reCj19250	2335	A/G	hkxhk	PRH75 DEAD box RNA helicase (PRH75)	–	0.00	AT5G62190.1
AX-115677965	9	34.123	947.48	100.0	MF_BC_CLC_contig_27478	273	A/G	nnxnp	Pre-mRNA-splicing factor 3	2139	0.00	AT1G28060.1
AX-115681104	9	34.475	99.99	100.0	reCj19314	126	T/G	hkxhk	Galactosyltransferase family protein	2081	0.00	AT5G62620.1
AX-115669347	9	34.475	99.99	100.0	reCj19314	166	T/C	nnxnp	Galactosyltransferase family protein	–	0.00	AT5G62620.1
AX-115676564	9	34.475	99.99	100.0	reCj19314	432	A/G	nnxnp	Galactosyltransferase family protein	–	0.00	AT5G62620.1
AX-115664995	9	34.475	99.99	100.0	reCj19314	859	T/G	hkxhk	Galactosyltransferase family protein	–	0.00	AT5G62620.1
AX-115679030	9	34.475	99.99	100.0	reCj19314	1623	A/G	nnxnp	Galactosyltransferase family protein	–	0.00	AT5G62620.1
AX-115665901	9	34.475	99.99	100.0	reCj19314	2587	T/G	nnxnp	Galactosyltransferase family protein	–	0.00	AT5G62620.1

### Putative causative genes

In the region where an LOD score ≥ 99.9 was detected on LG9, the 11 loci derived from the five isotigs near the significant QTL explained 100% of the phenotypic variation. The *A. thaliana* homologs of these isotigs in the TAIR10 database are shown in Table 1. These putative causative genes for the male-sterile trait have been reported as causative genes of male sterility in other species [41–46].

As one example, pre-mRNA splicing plays a role in removing introns from nuclear genes to generate functional mRNA in most of eukaryotes, regulating important developmental mechanisms. In rice, temperature-sensitive splicing (controlled by a *cis* splicing cite, small nuclear RNA, *trans* pre-mRNA splicing protein and SR protein via a complex cell signaling pathway) is thought to be caused by photoperiod and temperature-sensitive genetic male sterility [41]. In *A. thaliana*, pre-mRNA-splicing factor 3 (AT1G28060.1; 33.4 cM; MF_BC_CLC_contig_27478) is a component of U4/U6 small nuclear ribonuclear protein particles, and encodes *RDM16*, which may indirectly regulate DNA methylation and other aspects of gene transcription [47]. Mutation of *RDM16* and insertion of the corresponding DNA fragment into the At1g28060 promoter of an *rdm16 ros1* mutant led to morphological defects of leaves and siliques [47]. DEAD-box proteins encode RNA-dependent ATPases or ATP-dependent RNA helicases, which mediate conformational changes with respect to spliceosome assembly and disassembly through all of the processes dealing with RNA including its synthesis, modification, cleavage and degradation [41, 48]. In male sterility of rice, abnormal programmed cell death during the degradation of tapetal cells is caused by disrupted *apoptosis inhibitor5* (*API5*), a nuclear protein that interacts with two DEAD-box ATP-dependent RNA helicases, API5-INTERACTING PROTEIN1 (AIP1) and AIP2, which regulate the expression of the cysteine protease gene *CP1* [42]. These observations support the genes related to splicing that were associated with the significant QTL as candidates for the causative gene for male sterility in our Japanese cedar population.

Genes related to cell wall components were also associated with the significant QTL. Glycosyl hydrolases (GHs) are involved in the metabolism of various carbohydrate containing compounds present in plant tissues, with a major function in metabolism of most cell wall polysaccharides [49]. GH family protein 17, which is involved in pollen wall development, is a candidate gene for rice cytoplasmic male sterility based on F_2_ fine-mapping [43]. In Arabidopsis, *excess microsporocytes1* (*ems1*)/*extrasporogenous cells* (*exs*) mutants, which produce excess microsporocytes at the expense of the tapetum, and a *dysfunctional tapetum1* (*dyt1*) mutant show decreased expression of GH17 compared with wild type [50]. The *dyt1* mutant is suggested to act downstream of *ems1*/*exs* [51]. Similarly, in Chinese cabbage, the expression of most GH family proteins is downregulated in male-sterile individuals [44]. The candidate genes in Table 1 also include a galactosyltransferase family protein gene sequence. In Arabidopsis, this protein (AT5G62620: hydroxyproline-O-galactosyltransferase) is involved in arabinogalactan protein function, associated with important roles in plant growth and development [52]. In maize, the loss of function of the causative gene *Male sterile 8* (*MS8*)*,* putatively encoding β-1,3-galactosyltransferase, leads to an abnormal phenotype in epidermal and tapetal cells [45], and a functional connection between arabinogalactan protein and *MS8* is suggested in early anther development [46].

These observations thus indicate each of these genes as candidates for the causative gene for male sterility in Japanese cedar. We will be comparing these genes in male-sterile and male-fertile individuals.

### Marker development and validation

**Fig. 7:**
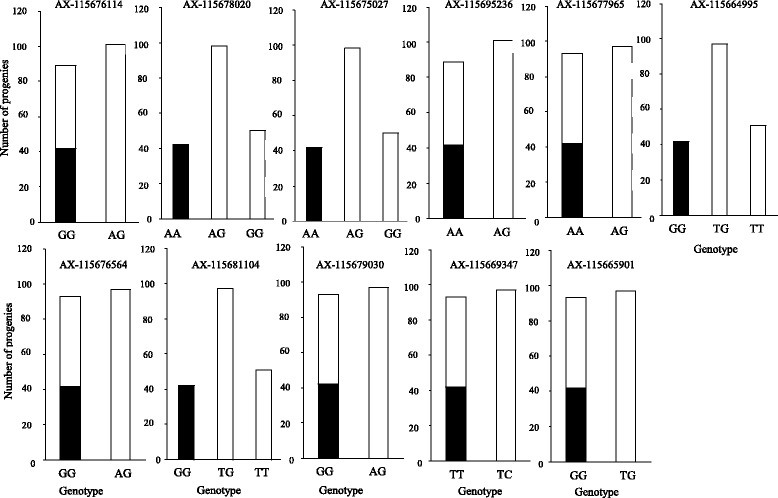
Genotypic effects of SNPs that explained 100% of phenotypic variation in significant QTLs. The black areas indicate male-sterile individuals, while the white areas indicate male-fertile individuals

**Fig. 8:**
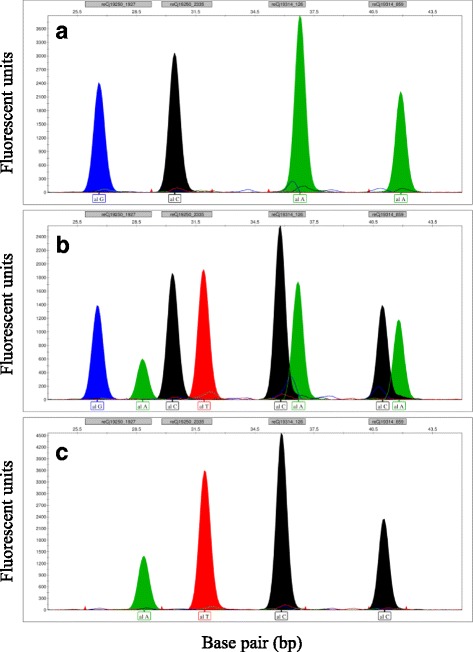
SNapShot genotyping electropherogram of four loci using the developed SNapShot markers. Representative examples of detection at the four SNP sites (four loci) of the contigs “reCj19250” and “reCj19314” by multiplex SNaPshot assay. **a** homozygous loci. **b** all heterozygous loci. **c** other homozygous loci (the opposite of A). The name and range of each locus is shown in the upper part of the X axis, and the nucleotide of the detected allele is shown in the lower part of the X axis. Nucleotides are represented by the following colors: A = green; C = black; G = blue; T = red

Since conventional tree breeding has been constrained by delays for evaluation of most economically important traits, which are expressed only at the adult stage, and hence by long breeding cycles, improvement of trees using MAS for selection of markers tightly associated with QTLs of important traits is extremely attractive [53–55]. However, in coniferous species, the identified QTLs for most economically important traits such as growth and wood properties generally explain only a small portion of phenotypic variation, so a MAS approach is not likely to be a successful strategy due to the complex genetic backgrounds for these traits [56–60], with the exception of some pest resistance genes [61, 62]. Because Japanese cedar pollinosis is a serious social problem in Japan, the male-sterile trait is mandated for breeding these trees. MAS is considered an effective and attractive solution, because the male-sterile trait is controlled by a recessive major gene [17, 18, 42, 46], and hence a definitive identification is expected if tightly linked markers are available. We identified one notable region for male sterility, and around it 11 marker loci derived from 5 isotigs. The relationship of the 11 marker locus genotypes with male sterility in the F_2_ population is shown in Fig. 7. Out of the 11 loci, all the male sterile individuals were homozygotes for one allele at four loci (AX-115678020, AX-115675027, AX-115664995, and AX-115681104), and the segregation ratio of the four loci was statistically consistent with Mendelian inheritance (1:2:1; chi-square test: *P* > 0.01). This finding indicated that these markers may also be able to discriminate heterozygotes (cryptic carriers). As male-sterile trees cannot be used as pollen donors, cryptic carriers with superior characteristics are essential for improving sterile trees and for establishing seed orchards, where sterile trees are pollinated by cryptic carriers, and sterile seedlings can be screened from the fertile cryptic carriers in the nursery using GA3 treatment to artificially promote induction of male strobili. To examine the potential of markers for precisely discriminating cryptic carriers, we developed four SNP markers (two markers for SNPs on reCj19250 (PRH75 DEAD-box RNA helicase) and two markers for SNPs on reCj19314 (galactosyltransferase family protein); Fig. 8, Table 2) that can be analyzed by a SNapShot assay, and genotyped 1082 plus trees and breeding materials selected from the Kanto breeding region. The allele and genotypic frequencies of the four markers are given in Table 3. The allele frequencies of nucleotide A in reCj19250_1927 and nucleotide T in reCj19250_2335, which are homozygous in ‘Sosyun’ were both 0.004, whereas the allele frequencies of nucleotide C in reCj19314_126 and reCj19314_859 in ‘Sosyun’ were 0.143 and 0.136, respectively (Table 3). The SNPs retained by ‘Sosyun’ at the two markers of reCj19250 can be regarded as very rare (≤0.01), and only seven plus trees were heterozygous at these two markers (Table 3). To confirm the screening potential of the two markers of reCj19250 as MAS markers outside of the mapping population, three of the seven heterozygous plus trees were crossed with ‘Sosyun’ or another heterozygous plus tree. The 63 progeny from the three families were genotyped using the SNaPshot assay and phenotyped for male sterility (Table 4, Additional file 7). Sterile progeny from all three families segregated, illustrating the effectiveness of the markers for screening cryptic carriers. The genotype arrays of the two markers for reCj19250 perfectly matched the male-sterile phenotype in all three families, whereas the markers for reCj19314 failed to discriminate sterile individuals precisely in one family. Thus, the reCj19250 markers are most suitable for MAS of male sterility.

**Table 2:** Development of SNaPshot primer set

Locus position	Forward/Reverse	Primer sequences	Amplicon size (bp)	Forward/Reverse	Extension primers	No.of nucleotides (bp)	Alleles (dyes) detected
				(Sense/Anti-sense)			
reCj19250_1927	Forward	5’-CTGGAAAGGTCATGTACTCTGC-3’	190	Forward	5′-1 T-AACATATGCGTTCACTACCCTGC-3’	24	G (blue), A (green)
	Reverse	5’-TCCCATCTACAGTAAGAGACATCC-3’		(Sense)			
reCj19250_2335	Forward	5’-GTTCTAGACGTGGCGGCAG-3’	180	Reverse	5’-TGCTCAATCCTGTACACAGTACCTGCAC-3’	28	C (yellow), T (Red)
	Reverse	5’-ACCTCGGAATGAACATTATGTGTTGAG-3’		(Anti-sense)			
reCj19314_126	Forward	5’-CGCTCTTCGTGTTGGCTTAG-3’	133	Reverse	5′-7 T-TCTCTGTGTGCCGACGAAACCGAAGT-3’	33	A (green), C (yellow)
	Reverse	5’-GGCTCAGAGATTACCACCTCG-3’		(Anti-sense)			
reCj19314_859	Forward	5’-GGTGGGCCTGTTTCCTATTCCG-3’	137	Reverse	5′-11 T-GGCCTCCATTGCAGACTTCTCAAACTG-3’	38	A (green), C (yellow)
	Reverse	5’-GCAGTTTCATCATCCATTACGGC-3’		(Anti-sense)

**Table 3:** Results of genotyping using SNaPshot primers

Locus	Genotype	Observed number	Frequency	Allele	Observed number	Frequency
reCj19250_1927	GG	1074	0.993	G	2155	0.996
	GA	7	0.006	A	9	0.004
	AA	1	0.001			
reCj19250_2335	CC	1074	0.993	C	2155	0.996
	CT	7	0.006	T	9	0.004
	TT	1	0.001			
reCj19314_126	AA	803	0.742	A	1854	0.857
	AC	248	0.229	C	310	0.143
	CC	31	0.029			
reCj19314_859	AA	814	0.752	A	1869	0.864
	AC	241	0.223	C	295	0.136
	CC	27	0.025

**Table 4:** Results of genotyping and phenotype in three crossed families

Phenotype and developed marker	Number of identified type		Crossed families		
			Plus-tree A (*Ms1/ms1*) × Plus-tree B (*Ms1/ms1*)	'Sosyun' (*ms1/ms1*) × Plus-tree B (*Ms1/ms1*)	'Sosyun' (*ms1/ms1*) × Plus-tree C (*Ms1/ms1*)
		Total number of Progeny	22	18	23
Phenotype	Fertile progeny		19	11	7
	Sterile progeny		3	7	16
reCj19250_1927	GG (*MS1/MS1*)		10	-	-
	GA (*Ms1/ms1*)		9	11	7
	AA (*ms1/ms1*)		3	7	16
reCj19250_2335	CC (*MS1/MS1*)		10	-	-
	CT (*Ms1/ms1*)		9	11	7
	TT (*ms1/ms1*)		3	7	16
reCj19314_126	AA (*MS1/MS1*)		10	-	-
	AC (*Ms1/ms1*)		9	11	23
	CC (*ms1/ms1*)		3	7	0
reCj19314_859	AA (*MS1/MS1*)		10	-	-
	AC (*Ms1/ms1*)		9	11	23
	CC (*ms1/ms1*)		3	7	0

This validation demonstrated that two of the markers could be used to i) screen for cryptic carriers outside of the mapping population and ii) precisely discriminate male-sterile individuals in the progeny. Thus, the markers we developed can realize the MAS approach for the first time in coniferous tree breeding.

## Conclusions

To discover causative genes associated with male sterility of ‘Sosyun’ and to develop MAS markers, we collected ESTs from several organs of Japanese cedar and carried out SNP discovery. More than half of the collected ESTs and SNPs were new, enlarging the genomic basis for genetic research on Japanese cedar. We constructed an EST-based high-density linkage map based on approximately 70,000 SNPs genotyped using the Axiom genotyping system and consisting of 3205 loci forming 11 linkage groups, with a mean distance between adjacent markers of 0.47 cM/marker, spanning 1492.8 cM. Using an F_2_ population, a significant QTL for male sterility was detected at 33.4-34.5 cM on LG9. The 11 marker loci associated with 5 isotigs explained 100% of the phenotypic variation, suggesting that one of the associated genes is causative. We developed two SNP markers aimed at MAS that distinguished individuals carrying the male-sterile trait with 100% accuracy, as well as individuals heterozygous at the male-sterile locus, even outside the mapping population. These markers should enable practical MAS for conifer breeding.

## Methods

### RNA extraction and sequencing

For construction of EST libraries, we used several organs: the cambium region in the dormant season, sapwood and heartwood, apical shoots, gibberellin (GA)-treated shoots, male strobili from male-sterile individuals, male strobili from male-fertile individuals, and roots of seedlings (Additional file 2). Total RNA was isolated from these organs using an RNeasy Plant Mini kit (QIAGEN, Gaithersburg, MD, USA) and RNA Isolation Reagent (Thermo Fisher Scientific, Waltham, MA USA). The quality of total RNA was assessed via an Agilent Bioanalyzer 2100 system (Agilent Technologies, Palo Alto, CA, USA). cDNA from each organ was synthesized from a mixture of RNA samples by nebulization, adaptor ligation, emulsion PCR and sequencing on a Roche 454 Genome Sequencer platform (Roche/454 Life Sciences, Branford, CT, USA) using FLX or Titanium technology, which was done at Hokkaido System Science Co., Ltd. (Sapporo, Hokkaido, Japan).

### Assembly of EST sequences from several organs and construction of reference sequences of *C. japonica*

The ESTs sequenced by the Roche 454 Genome Sequencer were trimmed of adapter sequences and poly(A/T) sequences by the cutadapt tool [63]. Then, low-quality sequences and short sequences (< 50 bp) were removed. Japanese cedar ESTs collected from the cambium region during the active season (DRA000525) [34] and shoots during the annual season (DRA001261) [35] were added following assembly. First, all of the reads from each library were assembled using GS De Novo Assembler version 2.8 software (Roche, Indianapolis, IN, USA) with default settings. Next, all of the reads from all libraries were assembled in the same way, and these assembled isotigs were regarded as reference sequences (Additional file 1).

To classify reference sequences according to organ, we compared reference sequences with isotigs of each library. Because reads were mapped, for example using the BWA software package [64], when only portions of ESTs showed high similarity, we selected a broader comparison by the BLASTN algorithm with default parameters, using reference sequences as queries and isotigs of each library as subjects. We regarded a reference sequence as expressed in an organ if the sequence showed high similarity (> 95%) to isotigs of the library from that organ and that region covered more than 95% of the length of the subject or query. Similarly, using BLASTN, reference sequences were compared with sequences from ForestGEN, an EST database of *C. japonica*.

### SNP discovery and SNP selection for axiom genotyping

For SNP discovery, resequencing to the reference sequences was performed for cambium of eight individuals (DRA00550 Experiment ID: DRX081263-70) and bulk samples of apical shoots of eight individuals (DRA00550 Experiment ID: DRX081272), shoots of eight individuals (DRA00550 Experiment ID: DRX081271), and male strobili of two male-sterile individuals (DRA00550 Experiment ID: DRX081274) and four male-fertile individuals (DRA00550 Experiment ID: DRX081273) using the Illumina HiSeq 2000 platform (Illumina, Branford, CT, USA) at Hokkaido System Science Co., Ltd. (Additional files 1 and 2). RNA extraction and the quality checks followed the same method used for EST library construction. Using a TruSeq RNA Sample Prep kit (Illumina), cDNA synthesis from an RNA sample from each organ, nebulization, adaptor ligation (including index tagging for individual recognition), bridge PCR and paired-end sequencing were performed on the Illumina HiSeq 2000 platform.

Reads sequenced on the Illumina HiSeq system were also trimmed of adapter sequences and poly(A/T) by cutadapt. Then, reads of each library were mapped to reference sequences by BWA [64] and SNPs were identified using SAMtools software [65] with default settings.

Because there are fewer available reference sequences for male strobili, we added more sequences from known ESTs of *C. japonica* and from de novo assembly for SNP detection in male strobili. The known ESTs were sequences from ForestGEN that did not show similarity to the reference sequences based on a BLASTN search. Illumina HiSeq reads from male strobili were de novo assembled by the CLC Genomics Workbench software package (QIAGEN) into more than 100,000 contigs (Mass Submission System ID: IABV01000001-01109392). About 20,000 contigs > 100 bp with average coverage > 10 reads/site were selected as references for SNP detection.

The 73,274 SNPs considered robust for genotyping were compiled into a set called Axiom_Cj70K_ver. 1 (Gene Expression Omnibus (GEO) Dataset GSE95616); we used SNPs obtained from Illumina HiSeq reads from a cambium and male strobilus library (Additional file 1). In Axiom genotyping, it is difficult to genotype SNPs adjacent to other SNPs. Hence, SNPs located within 20-50 bp of other SNPs were removed. SNPs with < 125 bp of sequence separating them from other SNPs were eliminated using a custom Perl script.

Next, we added more SNPs to Axiom_Cj70K_ver. 2 (GEO GSE95618), from Illumina HiSeq sequence reads of cambium of eight individuals, male strobili of two male-sterile individuals, male strobili of four male-fertile individuals, shoots of eight individuals, bulk samples of apical shoots of eight individuals and Roche 454 reads from GA-treated shoots (DRA00550 Experiment ID: DRX081260), sapwood and heartwood (DRA00550 Experiment ID: DRX081261) and roots (DRA00550 Experiment ID: DRX081262), which were also assembled into reference sequences, mapped to the reference sequence and used to identify SNPs (Additional file 5). As done for Axiom_Cj70K_ver. 1, SNPs located within 20-50 bp of other SNPs were removed. The surrounding sequence of the 53,378 SNPs was confirmed in Axiom_Cj70K_ver. 1 and 27,166 of these SNPs were compared to eliminate redundancy.

### Genotyping

Genomic DNA was extracted from progeny, parents and grandparents of the F_2_ population using a DNeasy Plant Mini Kit (QIAGEN). We used SNP markers obtained by resequencing to the reference sequence, which consisted of isotig sequences from next-generation sequencing data collected from various organs at several developmental stages and in different seasons using the Roche GS-FLX system. We also used two markers (cj_gSNP01452, cj_gSNP0438) reportedly in the vicinity of *ms-1* [17]. In total, 73,638 SNPs were used for genotyping using the Affymetrix GeneTitan system as described in the manufacturer’s guide. The SNP genotyping data were divided into six categories according to their clustering performance with respect to Axiom quality control criteria: (i) polymorphic high resolution, where the SNPs were clustered at a higher resolution, including at least two minor alleles; (ii) monomorphic high resolution, where the SNPs were clustered into one group except for the presence of a minor allele in two or more samples; (iii) no minor homozygote, where the SNPs were clustered into two groups except for the presence of a minor homoallele in some samples; (iv) call rate below threshold, where the genotype call rate was < 97%; (v) off-target variant, where atypical cluster properties arose from variants in the SNP flanking region; and (vi) other, where the SNP did not pass quality control. We selected the genotyping categories in (i) polymorphic high resolution and (iii) no minor homozygotes except a monomorphic allele in both parents of the progeny.

SSR markers covering 11 linkage groups of Japanese cedar have been reported [19], and 40 SSR markers were used as anchors for the genetic linkage map in this study. The genotype of each DNA sample was scored using 31 pairs of existing microsatellite primers [9–11, 19, 66]. Multiplex PCR with three or four SSR primer pairs was performed using a Multiplex PCR Kit (QIAGEN, Hilden, Germany), with 2× QIAGEN multiplex PCR master mix, 0.25 μM each primer pair, and 40 ng genomic DNA in a total volume of 10 μl. Amplification was performed in a Veriti thermal cycler (Thermo Fisher Scientific) using an initial denaturation step at 95 °C for 15 min, followed by 30 cycles of denaturation at 94 °C for 30 s, annealing at 57 °C for 1.5 min, and extension at 72 °C for 1 min, with a final extension at 60 °C for 30 min. PCR product (1 μl) was mixed with 0.2 μl GeneScan 500 LIZ size standard (Thermo Fisher Scientific) and 9.8 μl of Hi-Di formamide (Thermo Fisher Scientific) prior to electrophoresis. The length of the amplified fragments was analyzed with an ABI 3130xl sequencer (Thermo Fisher Scientific) and alleles were scored with GeneMapper v5.0 software (Thermo Fisher Scientific). In total, 73,678 markers were used for genotyping (Fig. 2).

#### Mapping population and traits

A set of 190 F_2_ individuals of 3-year-old trees from a cross between the male-sterile variety ‘Sosyun’ and two wild-type plus trees, Higashikamo7 and Kajikazawa6, was used for genetic linkage mapping and QTL analysis (Fig. 4). Male-sterile individuals were expected to make up one-fourth of the F_2_ mapping population. Harvested needles were immediately frozen in liquid nitrogen in the field, and then stored in the laboratory at − 80 °C for later DNA extraction. The male-sterile trait was evaluated by observing pollen collected from male strobili sprayed at a distance of ~ 30 cm from selected branches with 100 ppm GA3 solution (Kyowa-Hakko, Tokyo, Japan) on 15 July 2004. Pollen was observed under a stereomicroscope in mid-December 2014.

#### Construction of genetic linkage map and QTL analysis

A genetic linkage map was constructed using JoinMap 4.1 software (Kyazma, Wageningen, Netherlands) [67]. The marker segregation data were rescored as CP (cross between two heterogeneously heterozygous and homozygous diploid parents) population data, and grouped by the logarithm of odds (LOD = 10.0) using JoinMap’s maximum likelihood mapping algorithm (which estimated the default mapping parameters) and the Kosambi mapping function provided by the software. To ensure the correct marker order, markers with missing genotypes were removed. When the markers on the genotype array of 190 F_2_ individuals were compared in the mapping population, some marker pairs showed an identical segregation pattern, presumably due to tight linkage. In such cases, we arbitrarily selected one marker from each identically segregating marker pair for linkage map construction. On the constructed map, each linkage group was numbered and the SSR locus order validated using Japanese cedar reference maps [17].

QTL analysis with multiple-QTL model mapping (which estimated the default parameters) was performed to identify QTLs for the male-sterile trait using MapQTL 6.0 software (Kyazma) [68]. The trait data were recorded with a binary code of fertile (trait value of 0) and male-sterile (trait value of 1). Graphical representation of linkage groups and QTLs was carried out using MapChart 2.3 software [69].

#### Marker development and validation

Using an established SNaPshot assay (Thermo Fisher Scientific), which extends primers by a single base, we validated the markers. In the target SNP of each EST sequence, a primer was designed immediately upstream of the SNP (Table 2). Multiplex PCR with some PCR primer pairs for the SNapShot assay was performed under the same conditions as SSR analysis. To remove any primers and dNTPs, 5.0 μl of the PCR products was treated with 2.0 μl of Exo-SAP-IT reagent (Affymetrix, Cleveland, OH, USA), followed by incubation at 37 °C for 30 min, 80 °C for 15 min to inactivate the enzyme. Single-base extension reactions were carried out in a 5.0 μl final volume containing 0.5 μl SNaPshot Multiplex Ready Mix (Thermo Fisher Scientific), 0.2 μM each primer, and 2.0 μl of the treated PCR products. Reactions were performed in a Veriti thermal cycler, followed by 25 cycles of denaturation at 96 °C for 10 s, annealing and extension at 60 °C for 30 s. Final extension products were treated with 1 U shrimp alkaline phosphatase (Affymetrix) and incubated at 37 °C for 1 h, followed by enzyme inactivation at 80 °C for 15 min. PCR product (1.0 μl) was mixed with 0.2 μl GeneScan 120 LIZ size standard and 9.8 μl Hi-Di formamide prior to electrophoresis. Capillary electrophoresis was performed on a 3130xl Genetic Analyzer using POP-4 (Thermo Fisher Scientific), and alleles were analyzed with GeneMapper v5.0 software.

First, 1082 plus trees and breeding materials in the Kanto breeding region were genotyped and screened for the recessive homozygous or the heterozygous trait at each locus with the developed markers. Three plus trees with the heterozygous trait were used in cross-breeding with ‘Sosyun’ or other plus trees with the heterozygous trait. Three families were developed from these crosses. Each of the progeny in each family was genotyped using the markers and its traits were evaluated.

## Additional files

Additional file 1: Summary of collected ESTs, assembly and SNP discovery in this study. (PPTX 56 kb)

Additional file 2: *C. japonica* EST sequencing and assembly summary. *BC: Backcross. (XLSX 29 kb)

Additional file 3: The size distribution of our reference sequences in some model plant species. (PPTX 147 kb)

Additional file 4: Venn diagram showing the overlap among isotigs in four organs for our reference sequences. (PPTX 49 kb)

Additional file 5: Summary of SNP discovery in *C. japonica*. (XLSX 31 kb)

Additional file 6: Summary of all mapped markers. (XLSX 475 kb)

Additional file 7: Results of genotyping and phenotype in three crossed families. (XLSX 33 kb)

## Abbreviations

API: Apoptosis inhibitor
EST: Expressed sequence tag
GH: Glycosyl hydrolase
LG: Linkage group
MAS: Marker-assisted selection
MS: Male sterile
QTL: Quantitative trait locus
SNP: Single nucleotide polymorphism
SSR: Simple sequence repeat
