# Supplementary material for: Identification of novel putative causative genes and genetic marker for male sterility in Japanese cedar (Cryptomeria japonica D.Don)
Source: BMC Genomics. 2018 Apr 23;19:277. doi: 10.1186/s12864-018-4581-5 (PMC5914023; doi:10.1186/s12864-018-4581-5)
Supplement: Supplementary file 1 — Summary of collected ESTs, assembly and SNP discovery in this study. (PPTX 56 kb) [file 12864_2018_4581_MOESM1_ESM.pptx]

## Slide 1
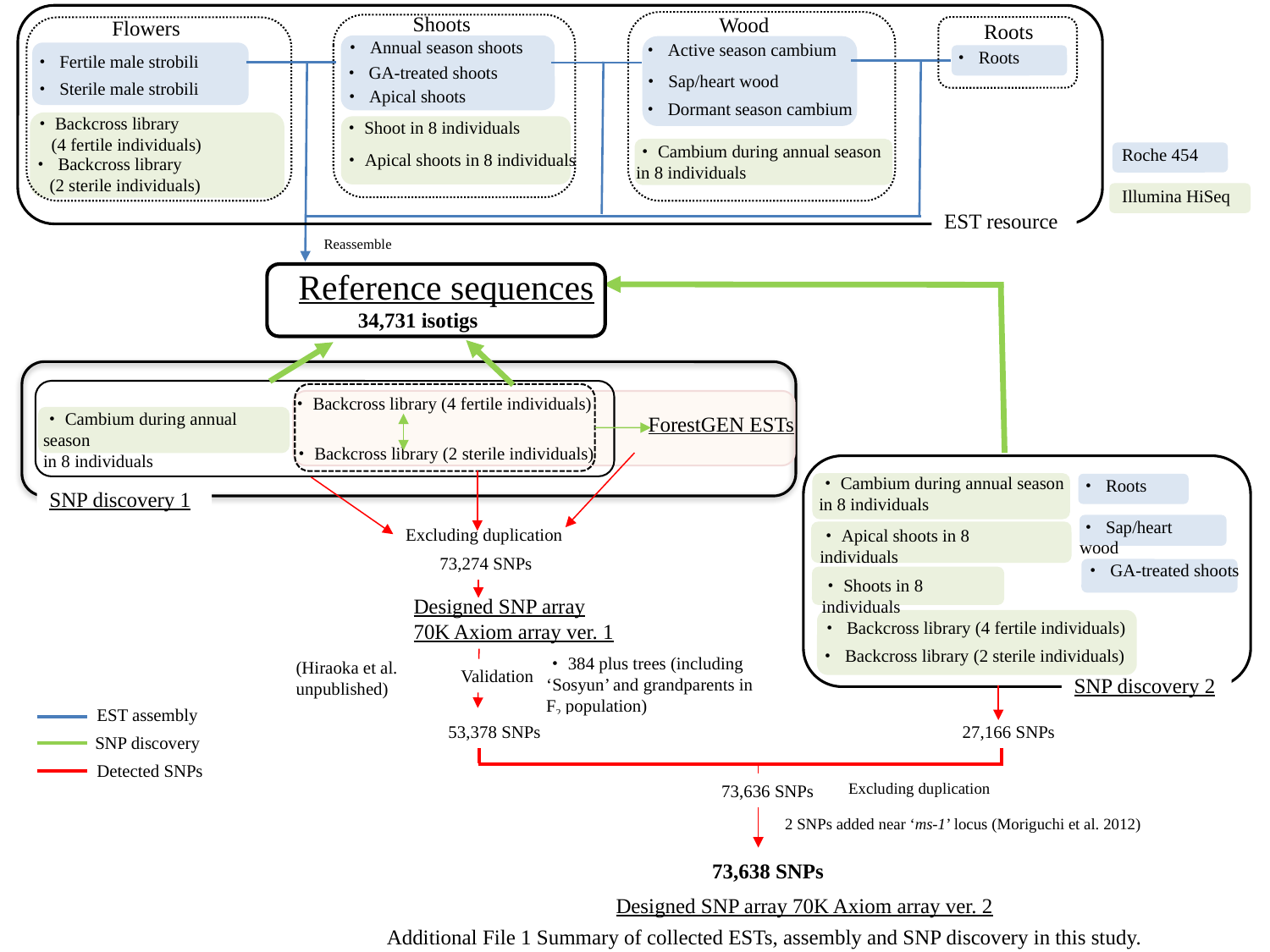

Shoots
Wood
Flowers
Roots
・ Annual season shoots
・ Active season cambium
・ Sap/heart wood
・ Dormant season cambium
・ Roots
・ Fertile male strobili
・ Sterile male strobili
・ GA-treated shoots
・ Apical shoots
・Backcross library
 (4 fertile individuals)
・ Backcross library
 (2 sterile individuals)
・Shoot in 8 individuals
・Cambium during annual season
in 8 individuals
Roche 454
・Apical shoots in 8 individuals
Illumina HiSeq
EST resource
Reassemble
Reference sequences
34,731 isotigs
・Backcross library (4 fertile individuals)
・Cambium during annual season
in 8 individuals
ForestGEN ESTs
・Backcross library (2 sterile individuals)
・Cambium during annual season in 8 individuals
・ Roots
SNP discovery 1
・ Sap/heart wood
Excluding duplication
・Apical shoots in 8 individuals
73,274 SNPs
・ GA-treated shoots
・Shoots in 8 individuals
Designed SNP array
70K Axiom array ver. 1
・ Backcross library (4 fertile individuals)
・ Backcross library (2 sterile individuals)
・384 plus trees (including ‘Sosyun’ and grandparents in F2 population)
(Hiraoka et al. unpublished)
Validation
SNP discovery 2
EST assembly
53,378 SNPs
27,166 SNPs
SNP discovery
Detected SNPs
Excluding duplication
73,636 SNPs
2 SNPs added near ‘ms-1’ locus (Moriguchi et al. 2012)
73,638 SNPs
Designed SNP array 70K Axiom array ver. 2
Additional File 1 Summary of collected ESTs, assembly and SNP discovery in this study.
