# Supplementary material for: Identification of novel putative causative genes and genetic marker for male sterility in Japanese cedar (Cryptomeria japonica D.Don)
Source: BMC Genomics. 2018 Apr 23;19:277. doi: 10.1186/s12864-018-4581-5 (PMC5914023; doi:10.1186/s12864-018-4581-5)
Supplement: Supplementary file 3 — The size distribution of our reference sequences in some model plant species. (PPTX 147 kb) [file 12864_2018_4581_MOESM3_ESM.pptx]

## Slide 1
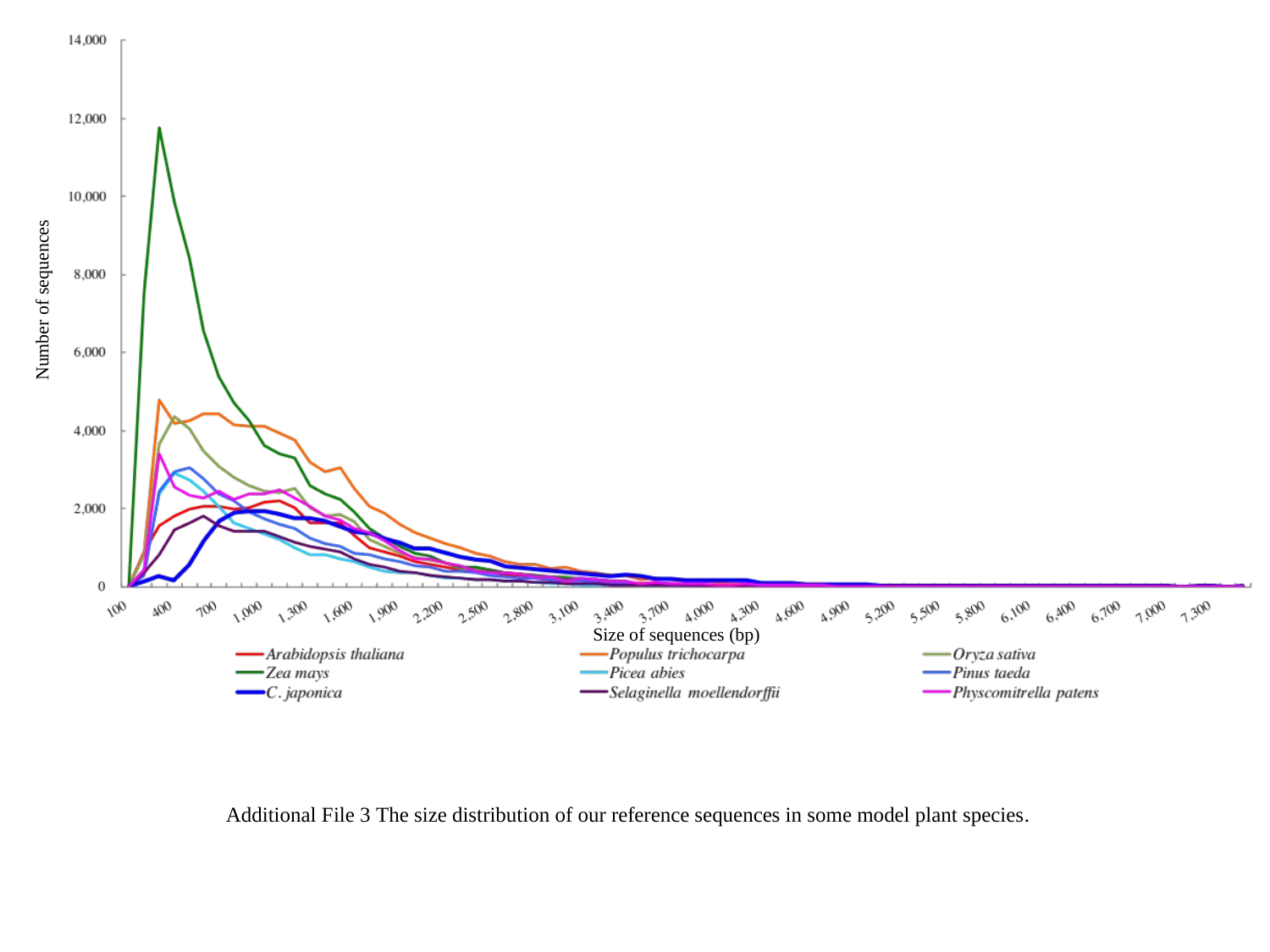

Number of sequences
Size of sequences (bp)
Additional File 3 The size distribution of our reference sequences in some model plant species.
