# Supplementary material for: Identification of novel putative causative genes and genetic marker for male sterility in Japanese cedar (Cryptomeria japonica D.Don)
Source: BMC Genomics. 2018 Apr 23;19:277. doi: 10.1186/s12864-018-4581-5 (PMC5914023; doi:10.1186/s12864-018-4581-5)
Supplement: Supplementary file 4 — Venn diagram showing the overlap among isotigs in four organs for our reference sequences. (PPTX 49 kb) [file 12864_2018_4581_MOESM4_ESM.pptx]

## Slide 1
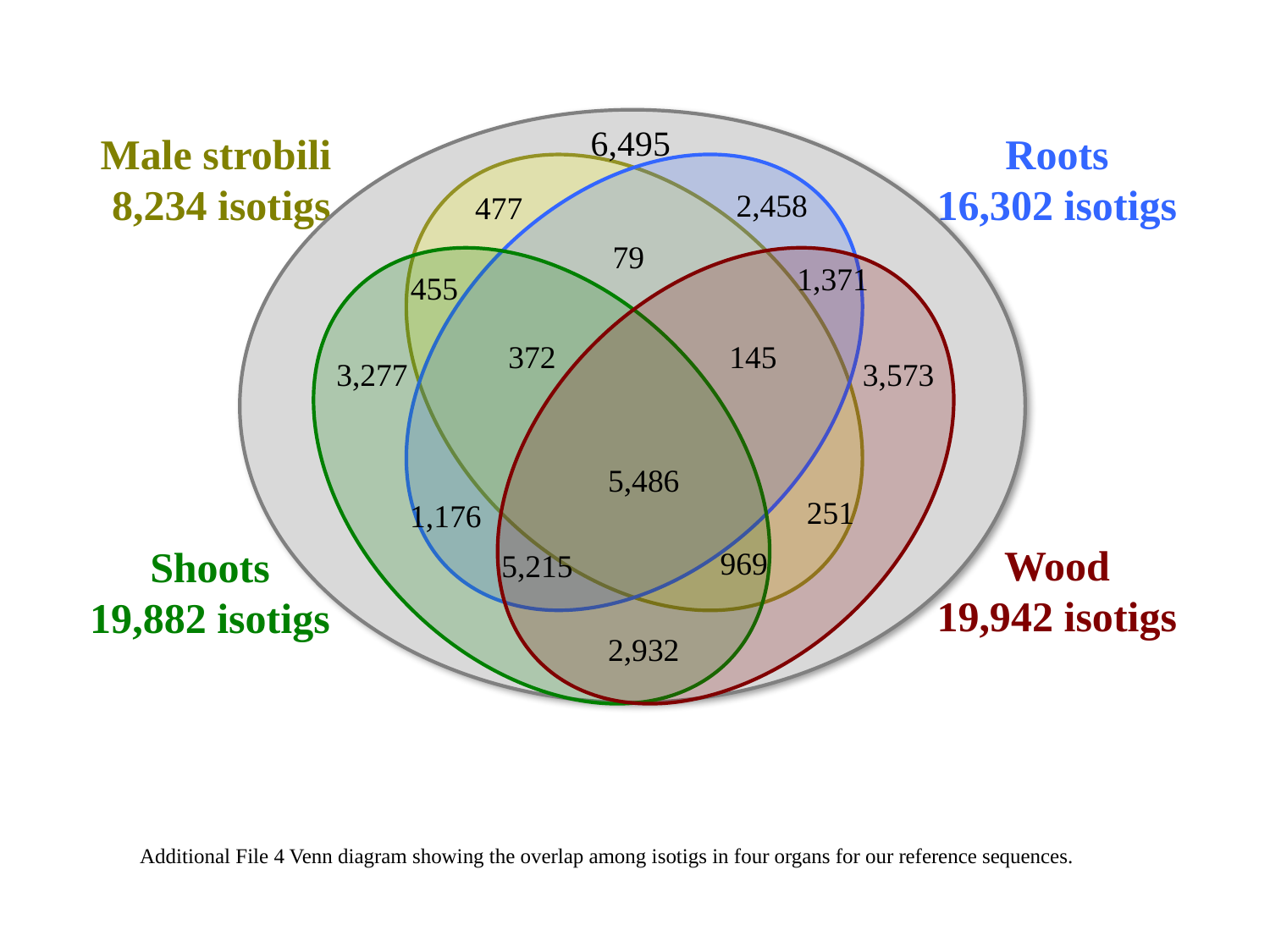

6,495
2,458
477
79
1,371
455
372
145
3,277
3,573
5,486
251
1,176
969
5,215
2,932
Male strobili
 8,234 isotigs
Roots
16,302 isotigs
Wood
19,942 isotigs
Shoots
19,882 isotigs
Additional File 4 Venn diagram showing the overlap among isotigs in four organs for our reference sequences.
